# Instant habits versus flexible tenacity: Do implementation intentions accelerate habit formation?

**DOI:** 10.1177/17470218221147024

**Published:** 2022-12-21

**Authors:** Tim van Timmeren, Sanne de Wit

**Affiliations:** 1 Habit Lab, Department of Clinical Psychology, University of Amsterdam, Amsterdam, The Netherlands; 2 Amsterdam Brain and Cognition, University of Amsterdam, Amsterdam, The Netherlands; 3 Department of Social, Health and Organisational Psychology, Utrecht University, Utrecht, The Netherlands

**Keywords:** Implementation intention, habit, strategic planning, goal-directed action, instrumental learning, outcome devaluation

## Abstract

Implementation intentions (strategic “if-then” plans) have been shown to support behaviour change. This may be achieved by mentally forming stimulus-response associations, thereby promoting habit formation. Does this deliberate attempt to instal “strategic automaticity” only offer advantages, or does it also come at the cost of reduced flexibility that characterises learnt habits? To investigate this, we tested healthy, young participants on a computerised instrumental learning task. Critically, we introduced implementation intentions (“if I see stimulus X, then I will respond”) versus goal intentions (“for outcome Z, I will respond)” during instrumental acquisition, and subsequently assessed behavioural flexibility in an outcome-revaluation test. In Experiment 1, we conducted a between-subjects manipulation of strategic planning, and in Experiment 2, a within-subject manipulation. We hypothesised that implementation intentions would lead to strong stimulus-response associations and consequently impair performance when the signalled outcome value changed and therefore required a different response, while benefitting performance when the outcome value (and required response) remained the same. We found that implementation intentions supported instrumental learning, but impaired test performance overall (most robustly in Experiment 2), irrespective of whether the signalled outcome value had changed. We argue that this general detrimental effect of implementation intentions on test performance is likely a consequence of their negative effect on stimulus-outcome learning. Our findings warrant caution when applying if-then plans to situations where the agent does not already possess perfect knowledge of behavioural contingencies.While implementation intentions may support efficient and fast behavioural execution, this may come at the expense of behavioural flexibility.

## General introduction

It is often challenging for people to achieve their goals, even if they have strong desires to reach them (Sheeran & Webb, 2016). The use of if-then plans (“If situation X arises, then I will perform response Y”), also known as implementation intentions, has been shown to promote this translation of intentions to actions (Gollwitzer, 2014; Gollwitzer & Sheeran, 2006). The beneficial effect of these if-then plans may in part be mediated by the formation of a mental stimulus-response (S-R) link (e.g., if I come home from work [S], then I will go jogging (R)), which may allow for the automatic activation of the specified response (Bieleke et al., 2021; Webb & Sheeran, 2007). This notion that the deliberate act of forming implementation intentions may prepare automatic processes is known as “strategic automaticity” or “instant habits” (Gollwitzer, 1993, 1999, 2014).

The idea that strategic planning could create an “instant habit” suggests that habits could be formed without having to execute the behaviour. This is intriguing, as it challenges one the main pillars of dominant habit models: namely that habits develop through *behavioural repetition* (Dickinson, 1985; Orbell & Verplanken, 2015; Wood & Rünger, 2016). Specifically, the influential Law of effect of Thorndike (1911) states that whenever behaviour is followed by a rewarding outcome, this will reinforce a stimulus-response (S-R) association between the behaviour that produced this outcome and the context in which it was performed. Thus, with repeated reinforcement of actions in the same context, S-R associations gradually become stronger. Could humans accelerate or even skip this gradual process by mentally forming the desired S-R link? In line with this possibility, previous studies have found that if-then plans are supported by several features of automaticity (Wieber et al., 2015), including efficient action initiation (Brandstatter et al., 2001; Gollwitzer & Brandstätter, 1997; Parks-Stamm et al., 2007) and the facilitation of action preparation and initiation even if cues are presented outside conscious awareness (Bayer et al., 2009). Furthermore, in line with the notion of strategic automaticity, forming an implementation intention has also been found to rapidly increase the self-reported automaticity of a novel flossing routine (Orbell & Verplanken, 2010). Taken together, previous research has provided support for the notion that implementation intentions enhance automaticity by transferring control of behaviour to contextual cues.

However, as pointed out by Orbell and Verplanken (2015), habits are not defined by automaticity alone. Another hallmark of habits is their inflexibility (Dickinson, 1985). According to dual-process accounts of associative learning (de Wit & Dickinson, 2009), the flexibility of behavioural control depends on the relative balance between a goal-directed and habitual process. Initially, actions are under control of the goal-directed process that is sensitive to the motivational value of their outcomes. However, when behaviour is followed by a rewarding outcome, this will lead to the gradual formation of S-R habits (Thorndike, 1911), and eventually these may exert dominant habitual control. In contrast to goal-directed actions, S-R habits are no longer driven by the anticipation and evaluation of the outcome, making them less flexible when goals change. The question arises whether implementation intentions similarly lead to inflexible behaviour when goals change, in line with the “instant habit” theory.

The question whether implementation intentions have the disadvantage of reducing behavioural flexibility was most directly addressed by a study by Legrand and colleagues (2017). To this end, they manipulated the costs associated with continuing to carry out a planned behaviour as specified by either an implementation or goal intention. They found that participants persisted in following their implementation (but not goal) intention when the associated costs were low (a time penalty or hearing white noise). In contrast, when the associated costs were high (a monetary penalty), they discontinued the behaviour regardless of plan format. From these results, the authors concluded that implementation intentions are “flexibly tenacious”: planned S-R mappings lead to perseverance as long as costs are bearable, but in the face of disproportionate punishment people will adjust the planned behaviour. However, from an associative learning perspective, this is also true for learnt habits. According to the aforementioned Law of Effect, an aversive outcome should gradually weaken the S-R association every time the behaviour is performed. The crucial distinction between goal-directed and habitual control is that the former is immediately and flexibly adjusted when the anticipated outcome is no longer valuable, in the absence of further experience with the instrumental contingency.

Therefore, the critical test of the relative balance between goal-directed and habitual control is whether performance is flexibly modulated by outcome value in the *absence* of outcome deliveries, that is, in extinction. To this end, the outcome-revaluation paradigm was developed four decades ago, initially in animals (Adams & Dickinson, 1981; Dickinson, 1985) but later also in humans (Watson & de Wit, 2018). The procedure involves an instrumental training phase, during which different responses are associated with different outcomes. After devaluing one of the outcomes (e.g., by satiation, taste aversion, or instruction), responding for the outcome is assessed in extinction. Goal-directed action control should allow one to immediately reduce responding for the devalued outcome, while a failure to flexibly refrain from responding is expected for behaviour under habitual control.

In this study, we therefore adopted an outcome-revaluation task to investigate whether implementation intentions lead to “instant habits.” Specifically, we employed a novel computerised outcome revaluation task, called the Symmetrical Outcome-Revaluation Task (SORT, Watson et al., 2022). In the first part of the task, participants learn to collect certain ice creams (outcomes) by pressing a response button upon seeing different ice cream vans (discriminative stimuli) to collect points. Importantly, during this instrumental training phase participants form either implementation or goal intentions. This is followed by the critical extinction test phase, during which some signalled outcome values change (i.e., *outcome revaluation*) and participants are again presented with the ice cream vans. They now have to quickly decide whether to respond or not, and a failure to flexibly adjust behaviour when the signalled outcome value is inconsistent with training provides a measure of the relative strength of habitual S-R control. This critical test phase thus allowed us to investigate whether learning to control one’s actions by implementations intentions is associated with reduced flexibility in the face of changing goals. The symmetrical design of this task, stemming from the inclusion of both valuable (promoting S-Go learning) and non-valuable outcomes (promoting S-NoGo learning), forces participants to initially learn to make responses based on the current value of the outcome. This is in contrast to outcome devaluation tasks (e.g., the slips-of-action task, de Wit et al., 2012) in which all outcomes are valuable, thereby not prohibiting participants to use a S-R strategy straight away. Another advantage of the symmetrical design is that it allows comparisons of congruent and incongruent test trials to be unconfounded by the nature of the correct response (Go or NoGo). We refer to Watson et al. (2022) for a more detailed discussion of the advantages of the symmetrical design, as well as potential differences between learning Go versus NoGo responses.

We preregistered the hypothesis that the use of implementation intentions (compared with goal intentions) would lead to increased reliance on previously formed S-R associations, as indicated by inflexible, habitual responding in the test phase and increased automaticity. We tested this prediction in two experiments, one using a between- and one using a within-subjects design.

## Study 1: between-subjects comparison of implementation and goal intentions

### Methods

All operationalisations, exclusion criteria and main hypotheses and analyses were preregistered on OSF (https://osf.io/uryfe). Any deviations from the preregistration are clearly indicated.

#### Participants

We aimed to collect a total of 30 usable datasets per group. Participants were randomly assigned to the implementation or goal intention group and recruited through the participant website of the University of Amsterdam website, flyers and word of mouth. The following inclusion criteria were used: being aged 16–35 years and not having previously participated in a previous study using this same task. All participants were native Dutch speaking college students. Data collection took place at the end of 2019. The study was approved by the Psychology Ethics Committee of the University of Amsterdam (2019-COP-10185) and performed in accordance with those guidelines. All participants gave written informed consent and received either course credit or financial compensation (15 euro) for their time (total 70 min). To motivate participants to perform well on the task, an additional €20 voucher was given to the participant with the highest score on the task.

#### Procedure

**Figure 1.**
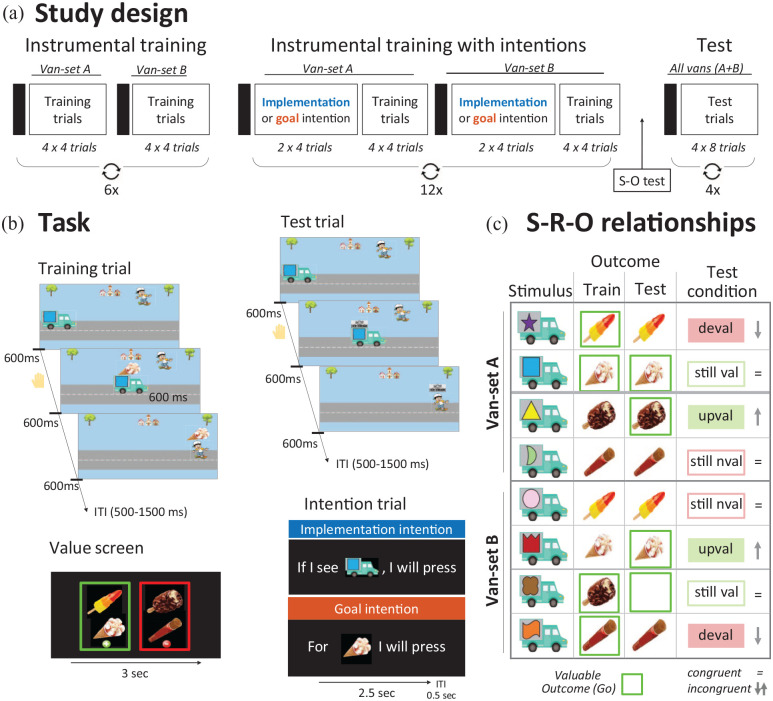
Study and experimental design. Participants were told they were playing a hungry skateboarder and their goal was to collect some ice creams (and not others) to earn points. (a) Participants first received instrumental training. Each block started with a value-screen (represented by a black rectangle), followed by a training block of 16 (four times four vans) trials (see b). Training blocks alternated between van-set A or B (see c) for a total of 12 blocks (six blocks per van-set). Training then continued with participants additionally using implementation intentions or goal intentions for another 24 blocks (12 per van-set). In Study 1, intentions were manipulated as a between-subject factor: one group was trained using implementation intentions and the other with goal intentions on both van-set A and B. In Study 2, participants used implementation intentions for van-set A and goal intentions for van-set B (within-subjects design). Finally, participants completed four test blocks in which all eight vans (van-set A and B) would appear intermixed and consequently for some vans the associated outcome-values changed compared with training. (b) Value screen: the outcome-value screen informs participants of which ice creams they should (in green) and should not (in red) collect at the start of each block. Train trial: participants had to decide whether or not to make a response within 600 ms, after which the ice cream was shown on the moving van. Then, the ice cream was shown (irrespective of a response) and the van continued to move right with the ice cream on top until leaving the screen (600 ms). Only if a response was made, the skater moved to down to collect the ice cream as the van approached the right-hand side of the screen and the skater and ice cream remained on the screen for another 600 ms. Test block: similar to train blocks but now vans had a banner on top instead of the ice cream, so no more feedback was given (i.e., nominal extinction). (c) An example overview of stimulus-outcome contingencies and associated values across different phases of the task. During training blocks, participants saw the first four (van-set A) or the last four ice creams vans (van-set B) during separate blocks (assignment of stimuli randomised). The contingencies between each ice cream and van remained consistent throughout the whole task. The value of the outcome was stable only during training, when participants learned by trial and error to respond for vans signalling valuable ice creams and withhold making a response for non-valuable ice creams. During the critical test phase, the associated outcome changed for half of the stimuli. For example, the first van always delivered a Rocket, which was valuable throughout training but no longer valuable during test (i.e., devalued). Shown here is an example of the contingencies in one of the four test blocks; across the test phase the correct response for each stimulus was equally often congruent and incongruent.

Participants performed a computerised instrumental learning task called the Symmetrical Outcome Revaluation Task (SORT) to investigate habit formation and expression. They were told that in this game they play a hungry skateboarder, and their goal was to collect ice-creams to satisfy their hunger and collect points by pressing the space bar. The participant who obtained most points at the end of the study received a €20 voucher. Four pictures of ice creams were used: a Cornetto, a Magnum, a Rocket ice lolly, and a soft serve ice cream. The task consisted of three phases: an instrumental training phase, an instrumental training phase with implementation and goal intentions, and an extinction test phase (see Figure 1).

#### Task and materials

##### Instrumental training without strategic planning

At the start of the task, participants were told that they should press the spacebar (Go response) to ice-cream vans with different logos superimposed. These discriminative stimuli (S) signalled which outcome (O) could be earned by pressing the spacebar. Some of the outcomes were worth points (valuable), and some led to subtraction of points (not valuable). When a valuable outcome was signalled, participants should press the spacebar (i.e., Go), but when a non-valuable outcome was signalled, they should refrain from pressing the spacebar (NoGo).

Prior to each block of instrumental training, participants were shown for 3 s which two ice creams should be collected (in green) and which two ice creams should be avoided (in red), called “value-screen” (Figure 1a). There were eight different vans that were divided over two sets (i.e., sets A and B; Figure 1b) and each ice-cream was associated with two different vans. Each training block contained one set of van stimuli and blocks alternated between van-set A (S1–S4) and van-set B (S5–S8). Two of the ice creams (O1 and O2) were always valuable during the set A blocks whereas the other two ice creams (O3 and O4) were always valuable during set B blocks. Participants were told to find out by trial and error which ice-cream truck delivered which ice-cream, and that the stimulus-outcome contingencies would remain the same throughout the whole task. The assignment of ice creams (O1–O4) to the vans (S1–S8) was randomised across participants. To familiarise participants with the general procedure, they first practised two blocks of training with different discriminative stimuli (scooters) and outcomes (pizza’s).

Each of the 12 blocks consisted of 16 trials, with each stimulus being shown four times per block. Trial order was randomised per eight trials, with each van being presented two times in the first and two times in the second half of a block. Each trial started with a 500–1000 ms intertrial interval (ITI), during which a road was depicted. One of the vans then moved from the left to right end of the screen (1200 ms total). We instructed participants that, to obtain an ice-cream, they should respond as quickly as possible and before the ice cream appeared (after 600 ms, halfway across the screen). Irrespective of the response, the associated ice cream was then shown on top of the van, which continued moving right until it exits (for another 600 ms). If no response was made within the response window, the ice cream was not collected and continued off the screen with the ice cream van. If a response was made, however, the skateboarder moved down to collect the ice cream and the ice cream would remain on the screen for an additional 600 ms. Participants did not receive direct feedback about the accuracy of their response (e.g., if they *correctly* responded for a valuable- or incorrectly for a non-valuable ice cream). We did this to promote goal-directed learning (i.e., based on stimulus-outcome relationships). Participants were instructed that they earned one point for collecting a valuable ice cream and lost one point for collecting a non-valuable ice cream. At the end of each block, participants did receive feedback about accuracy (separately for valuable and non-valuable outcomes), the number of late responses and the number of points collected in that block for 5 s. We did this to give them a general impression of how they were performing and motivate them to improve. After six blocks, participants were granted a 30 sec break (or whenever they pressed the space bar).

##### Instrumental training with strategic planning

After the first 12 blocks of instrumental training, participants were told that they would continue training for another 24 blocks. As before, each block started with a value instruction screen, that showed participants which ice creams were valuable or non-valuable. But this time, before preceding to the instrumental training block, participants additionally rehearsed action plans. Half of the participants received implementation intentions, indicating for which ice cream *van* they should make a response, formulated in the form “If I see [picture of an ice cream truck] then I WILL (NOT) press.” The other half received goal intentions, indicating for which *ice cream* they should or should not make a response, formulated as “For [picture of an ice cream], then I WILL (NOT) press” (Figure 1b). Specifically, each training block started with rehearsal of four different intentions repeated twice (order randomised) for 2500 ms, followed by a 500-ms intertrial interval. Participants were asked to read the specified intentions out loud. Subsequently, the instrumental training block started with the stimuli and outcomes that corresponded to the formulated action plans. These instrumental training blocks were identical to the previous phase without strategic planning.

Before starting the real training (with vans and ice creams), participants first practised one block with the scooters and pizzas (also used at the beginning of the task) and were presented with an overview of all eight vans to ensure that they were able to quickly name the ice cream vans when reading out loud the intentions.

##### Test of stimulus-outcome knowledge

At the end of training with intentions, participants were asked about their explicit knowledge of the stimulus-outcome (S-O) contingencies and confidence. Each ice cream van was presented, and participants had to select the signalled ice cream. Subsequently, they were asked to indicate how confident they were about their decision using the mouse, with the scale running from 0 to 100. A composite score reflecting S-O knowledge was calculated by multiplying the average number of correctly reported S-O contingencies with the confidence measure.

##### Outcome-revaluation test phase

At the start of the test phase, participants were told that the final phase would be more challenging because all eight ice cream vans would appear intermixed during each block, and a banner would be placed on top of the ice cream vans that prevented them from seeing the associated ice cream. They were also informed that the ice creams trucks continued to deliver the same ice creams as during training. We instructed them to pay extra attention to the value-screens because some ice cream vans that previously always delivered valuable ice creams would now lead to a reduction of points (i.e., devalued trials) and vice versa (i.e., upvalued trials). Again, participants were first familiarised with the test-phase procedure by practicing one block with the scooters and pizzas.

The test phase was similar to the training phase, with three important differences. First, as intention blocks were no longer presented, value screens were again shown at the start of each block. Second, the ice creams that were shown on top of the van during training were now replaced by a banner (i.e., nominal extinction) to prevent new learning to occur. Finally and crucially, all eight vans were presented intermixed (four times per stimulus) in each block. Consequently, for half of the vans in each block, the value of the signalled outcome was congruent with training value, but incongruent with the value they had been trained on for the other half. For example, if during training O1 was always valuable in blocks with van-set A but not block van-set B, then S1 signalled a Go response and S5 a NoGo response. If during test O1 was *not valuable*, this was congruent with the trained NoGo response for S5 but *in*congruent with the trained Go response signalled by S5—the outcome was devalued, and the response should be inhibited. This resulted in four different conditions for each intention-type: “still valuable”—the outcome signalled by this stimulus was always valuable during training and also valuable during this test block (i.e., “value-congruent” with training); “upvalued”—the outcome signalled by this stimulus was not valuable during training but valuable during this test block (i.e., incongruent); “still not valuable,”—the outcome signalled by this stimulus was not valuable during training and also not valuable during this test block (i.e., congruent); and “devalued trials”—the outcome signalled by this stimulus was always valuable during training but not valuable during test (i.e., incongruent).

#### Preregistered behavioural data analysis

Behavioural data analyses were performed using IBM SPSS Statistics 25 for Mac (IBM, New York, NY, USA) for frequentist statistics and JASP version 0.16.3 (JASP Team, 2018) for Bayesian statistics. An annotated .jasp file including the full analysis pipeline is available at https://osf.io/qpaxs/.

Although we preregistered to use percentage response rates as the main outcome variable, we have since then realised that percentage accuracy provides a better measure of performance, and this is now our consistent approach to analysing the data of this novel task, used in our recently published (first) study using the symmetrical outcome-revaluation task (Watson et al., 2022). Accuracy and response rates are the same on valuable trials, but they differ on not-valuable trials when making a response (i.e., higher response rate) is inaccurate (i.e., lower accuracy). When using response rates, this creates unnecessary interactions with value during both training and test, which are eliminated when accuracy is used (because any effects of intention on performance will be in the same direction for both Go and NoGo). The hypothesised (and preregistered) three-way interaction between test-value, value-congruence and intention-type thus reduces to a two-way interaction between value congruence and intention-type which in turn halves the number of preregistered hypotheses in the analysis.

For data analysis purposes, the between-subjects training data were collapsed across blocks of two, referred to as block sets. To assess that learning had taken place over the first part of the training without intentions, accuracy was analysed using a 2 x 2 x 6 mixed analysis of variance (ANOVA) with within-subject factors value (valuable or non-valuable) and block-set (1–6) and with intention-type (implementation or goal-intention) either as between- or within-subjects factor in Study 1 or 2, respectively. The second part of training was analysed using a similar repeated measures/mixed ANOVA, as an additional factor. Response times were analysed with similar analyses of variance (ANOVAs), but for valuable trials only.

For the test phase, data were analysed using a 2 × 2 × 2 mixed ANOVA with the factors test-value (valuable or non-valuable during test) and congruency (congruent or incongruent with value during training) as within-subject factor, and intention (implementation or goal-intention) as between-subject factor in Study 1 and within-subject factor in Study 2. Thus, for each intention-type, there are four conditions: still valuable trials (valuable, congruent), upvalued trials (valuable, incongruent), still not valuable trials (non-valuable, congruent) and devalued trials (non-valuable, incongruent). First, we expected a main effect of congruence (higher accuracy on congruent vs incongruent trials), indicating a habitual failure to adjust a learned response. Second, we hypothesised that there would be an interaction with intention type such that the use of implementation intentions would lead to lower accuracy for incongruent outcomes specifically.

In the case of violations of sphericity, we report Greenhouse–Geisser corrected degrees of freedom and *p*-values. We report partial eta squared (*η*_p_^2^) for the ANOVAs and Cohen’s d for t-tests are reported as estimates of effect sizes, in addition to 95% confidence intervals for *t*-tests. For the main variables of interest, we will also report non-significant higher-order interactions. As preregistered, we additionally report the Bayes Factors (BF) that result from corresponding Bayesian analyses for the main analyses of interest. For null results (*p* > .05), we report the BF_excl_, which quantifies evidence in favour of *excluding* a certain predictor in the model. For significant results (*p* < .05), we report the BF_incl_, which quantifies evidence in favour of *including* a certain predictor (e.g., a factor in the ANOVA) in the model (and is identical to 1/BF_excl_). The reported Bayes factors result from the analysis of effects comparing all models (not only matched models; see van den Bergh et al. (2020) and including random slopes which is more similar to the frequentist model specification (van den Bergh et al., 2022). Bayes factors are interpreted according to Wetzels et al. (2011; Table 1), with BF between one and three reflecting anecdotal support, BF larger than three reflecting substantial support and BF larger than ten reflecting strong support. In all Bayesian analyses, JASP’s default priors were used.

### Results

**Figure 2.**
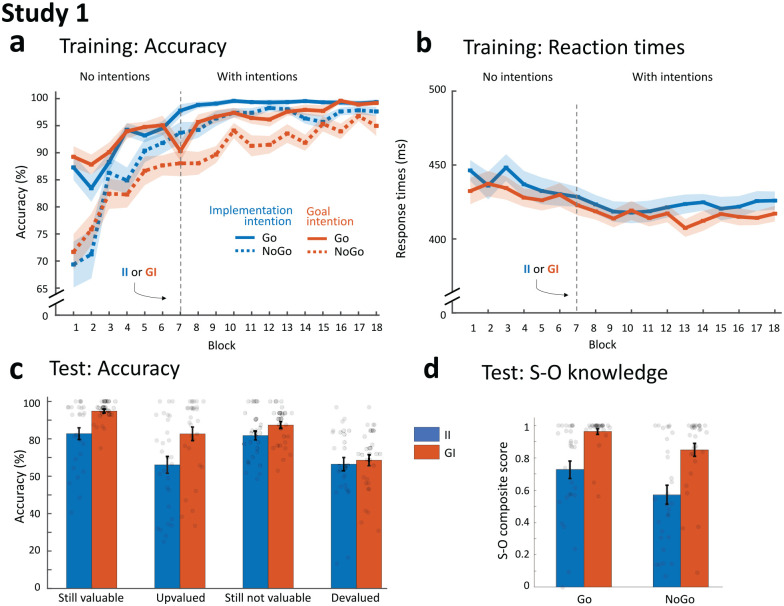
Main results of Study 1. (a) Mean accuracy across training. Participants learned to successfully respond for stimuli associated with a valuable outcome (Go) and to withhold making a response for stimuli associated with a non-valuable outcome (NoGo), as reflected by increasing accuracy rates. After six blocks of regular training (black dotted line), some participants continued training with implementation intentions while others used goal intentions. Accuracy was significantly higher initially when using implementation intentions, but towards the end of training performance was almost perfect for both groups. (b) Mean reaction time across training. Participants became faster over training, with no significant differences between the groups. (c) Mean accuracy during the test phase, when for some stimuli the associated outcome changed in value (and thus response) compared with training (upvalued [Go] or devalued [NoGo]; see Figure 1c) and participants had to flexibly update their responses accordingly. For other stimuli, the associated value and response stayed the same: still valuable (Go) or still not valuable (NoGo). First, across all participants there was lower accuracy for incongruent compared with congruent trials, reflecting inflexibility as a consequence of learned S-R contingencies during training. In addition, participants in the implementation intention group were less accurate when discriminative stimuli signalled a still valuable or upvalued outcome compared with the goal intention group. (d) Mean S-O knowledge. At the end of training, participants in the implementation intention group had significantly worse knowledge of the stimulus-outcome associations (i.e., which van delivered which ice cream) than participants trained with goal intentions. Knowledge for trucks delivering non-valuable ice creams (NoGo) was also worse than for valuable (Go) ice creams. Shaded regions (in A and B) and error bars (in C and D) represent standard error of the mean. II: implementation intention group; GI: goal intention group; RT: reaction time; S-O knowledge: Stimulus Outcome knowledge.

Data to recreate all analyses as well as the full analysis pipeline with output (in JASP) are available at https://osf.io/qpaxs/. Training results without and with strategic planning are shown in Figure 2a and b.

#### Participants

Our preregistered target sample was 30 participants per group. No participants were excluded based on the training criterion (<80% accuracy in the final block-set), while 10 participants (six from implementation intentions group) were excluded because of the preregistered test criterion (<25%) on upvalued trials), leaving a total of *n* = 29 (23 females) in the implementation intention group (mean age = 20.6, *SD* = 2.5) and *n* = 31 (22 females) in the goal intention group (mean age = 20.3, *SD* = 2.2).

##### Instrumental training without strategic planning

A mixed ANOVA on accuracy with factors Block-set, Value and Group showed that, as expected, participants learned to make correct responses to the discriminative stimuli (main effect of block-set: *F*_3.8,222.2_ = 32.68, *p* < .001 *η*_p_^2^ = .36). Furthermore, a significant main effect of value (*F*_1,58_ = 24.50, *p* < .001, *η*_p_^2^ = .30), driven by overall better performance on Go trials, was superseded by an interaction with block-set (*F*_3.5,20.9_, = 7.74, *p* < .001, *η*_p_^2^ = .12). This reflected that, although accuracy was significantly higher on Go than on NoGo trials during all block-sets, this difference was most pronounced early in training (see Figure 2a). As expected, both intention groups performed equally well in these pre-intention training blocks (all effects of group *p* > .33). Groups also did not differ in reaction time (*p* = .3), but participants became faster over training (on Go trials, Figure 2b), as suggested by a borderline-significant effect of response time (*F*_4.2,249.5_ = 2.36, *p* = .051, *η*_p_^2^ = .04).

##### Instrumental training with strategic planning

In the remainder of training with intentions, the mixed ANOVA (Block-set by Value by Group) showed that participants continued to improve performance (main effect of block: *F*_6.6,382.0_ = 9.10, *p* < .001, *η*_p_^2^ = .14) and respond more accurately for Go compared with NoGo trials (main effect of value: *F*_1,58_ = 34.8, *p* < .001, *η*_p_^2^ = .38). Moreover, a significant interaction between intention-group and block (*F*_6.6,382.0_ = 2.91, *p* < .001, *η*_p_^2^ = .05) was driven by better performance for the implementation relative to goal intentions group early in training (block-sets 7–14: *p* < .01); a difference that was non-significant from block-set 15 onwards (block-set 15: *F*_1,58_ = .83, *p* = .37, *η*_p_^2^ = .01; final block-set: *F*_1,58_ = 1.87, *p* = .18, *η*_p_^2^ = .03). No significant effects of block, intention or their interaction were seen on response times (all *p* > .11).

To examine whether the effect of intention-type early in training was due to a beneficial effect of implementation intentions or detrimental effect of goal intentions, we explored the difference between the final training block without (block-set 6) and the first block of training with intentions (block-set 7). Paired t-tests revealed evidence for both: the group that used if-then planning showed a trend towards better performance on Go trials when intentions were introduced (*t*_28_ = –1.81, *p* = .08, *d* =–.34, 95% CI = [–.71, .04]), while goal-intentions had the opposite effect (*t*_30_ = 2.18, *p* = .04, *d* = .39, 95% CI = [.02, .75]). This pattern was specific to valuable trials; no significant effect of intention was seen on NoGo trials (all *p* > .47).

##### Symmetrical outcome-revaluation test

Test phase was analysed with a mixed ANOVA with factors Test-value, Congruence and Group. As can be seen in Figure 2c, there was a strong main effect of congruence (i.e., worse performance on incongruent trials compared with congruent with training value; *F*_1,58_ = 68.47, *p* < .001, *η*_p_^2^ = .54), reflecting the effect of learned S-R mappings. However, the anticipated interaction with intention group, which would imply reduced flexibility as a consequence of training with implementation intentions, was not significant (*F*_1,58_ = .02, *p* = .90, *η*_p_^2^ = .00). Bayesian analysis indicated anecdotal evidence against this interaction (BF_excl_ = 1.72). Nevertheless, the use of if-then planning during training did have an effect on test performance, as revealed by a significant Test-value by Group interaction (*F*_1,58_ = 6.79, *p* = .01, *η*_p_^2^ = 0.11, BF_incl_ = 6.75). Separate mixed ANOVAs for cues signalling outcomes that were valuable (still valuable and upvalued) and non-valuable (still not valuable and devalued) during test revealed that accuracy was higher for the group trained with goal compared with implementation intentions on valuable Go-trials (*F*_1,58_ = 14.80, *p* < .001, *η*_p_^2^ = 0.20) with very strong Bayesian evidence (BF_incl_ = 66.9), but not on non-valuable NoGo trials (*F*_1,58_ = 1.38, *p* = .25, *η*_p_^2^ = .02), with anecdotal evidence against an effect of intention (BF_excl_ = 2.10).

A similar analysis of response times showed a significant interaction between test-value and congruence (*F*_1,49_ = 47.66, *p* < .001, *η*_p_^2^ = .49) driven by slower responses for upvalued compared with still valuable trials, but faster responding for (mistakenly) responding on devalued compared with still not valuable trials, in line with the idea that responses on devalued trials are “slips of actions.” However, no main or interaction-effects with intention-type were observed (all *p* > .48).

##### S-O knowledge

S-O knowledge (Figure 2d) was significantly lower for the group trained with implementation relative to goal intentions (*F*_1,58_ = 21.9, *p* < .001, *η*_p_^2^ = 0.27) and stimuli associated with non-valuable versus valuable outcomes (*F*_1,58_ = 17.3, *p* < .001, *η*_p_^2^ = 0.23).

Based on the significant effects of implementation intentions on test performance and S-O knowledge, we decided to run additional exploratory analyses to test their relationship. Nonparametric correlations (using Kendall’s tau, as the data were not normally distrusted) between test-accuracy (mean across four conditions) and S-O knowledge (mean composite score across all eight S-O contingencies) showed that these were positively related (implementation intentions: *r*_τ_ = .52, *p* < .001, 95% CI = [.39, .65]; goal intentions: r_τ_ = .34, *p* < .001, 95% CI = [.39, .65]).

### Interim conclusions Study 1

In line with the notion that implementation intentions increase efficiency, we found that these if-then plans resulted in higher accuracy compared with goal intentions during early training, but only when planning to make a response (i.e., on Go trials). Still, by the end of instrumental training with intentions, discriminative performance was at an equal (near-perfect) level independent of the intention used. However, the use of implementation intentions still impaired performance during the test phase, at least during the Go trials: when discriminative stimuli signalled a still valuable or upvalued outcome, the group that used implementation intentions performed worse than the group using goal intentions. While this detrimental effect of implementation intentions indicates reduced behavioural flexibility, this is not readily explained in terms of stronger S-R associations, as the effect was observed across congruent and incongruent (Go) test trials. Instead, this impairment may be related to the impact of implementation intentions on the acquisition of S-O knowledge. When offered S-R plans, people may rely on these at the expense of learning about the full three-term contingencies between antecedent cues, actions and outcomes. However, it is still puzzling why the effect would be specific to Go test trials, as S-O knowledge for implementation intentions was worse for both Go and NoGo stimuli. It could be that with lower S-O knowledge participants become more hesitant to make Go responses but that should also be reflected by the opposite pattern for NoGo trials, which was not the case.

Next, we aimed to replicate and extend these results by manipulating intentions as a within-subject factor, while minimising random (between-subjects) noise. A potential downside of manipulating implementation and goal intentions within-subjects is that this may cause transfer effects: participants may start using implementation intentions on goal intention trials (and vice versa), which would mask any difference between intentions. However, if we do replicate the observed detrimental impact of implementation intentions, this would provide a strong demonstration of the replicability and robustness of the results of Study 1. Conversely, it could also be that the detrimental effects of if-then planning on test-performance are (partly) masked by a carry-over effect from training to test: participants who were trained with implementation intentions may have also applied this strategy to support behaviour change during the test phase. That would give them an advantage compared with the goal intention group, who did not have this strategy readily at their disposal during test. Using a within-subject design may in this case reveal a stronger detrimental effect of implementation relative to goal intentions on test performance, as both conditions can now benefit equally from applying that strategy to support test performance.

## Study 2: within-subject comparison of implementation and goal intentions

### Methods

The methods of Study 2 were identical to Study 1, except for the way that intentions were manipulated. Instead of investigating the effects of implementation intentions using a between-subjects design, that is, by training participants with either implementation or goal intentions on all eight stimuli, participants were now trained using implementation intentions for one half of the stimuli (set A), while the other four stimuli (set B) were trained using goal intentions (starting-intention counterbalanced across participants). Consequently, despite the total number of training blocks being equal participants in the within-subjects design received only half the number of training blocks per intention-type (12 instead of 24 blocks).

### Results

Again, all data to recreate the analyses and the full pipeline with output (in JASP) are available at https://osf.io/qpaxs/.

#### Participants

Because of an error in the task-script, the data of the first 10 participants were not usable and removed from further analysis. Of the remaining 38 participants, no participants were excluded based on the training performance criterion (<80% in the final block-set), but eight participants were excluded because of low accuracy (<25%) in the test-phase on upvalued trials trained with goal intentions. The purpose of this exclusion criterion is to ensure that all participants included in the analysis understood the test-phase instructions and updated their performance accordingly (at least to some extent). Therefore, we exclusively used performance on goal intention as an exclusion criterion and not implementation intention, which is the manipulation of interest. The final 30 participants (28 females) had a mean age of 19.7 years (*SD* = 2.1).

#### Instrumental training without strategic planning

**Figure 3.**
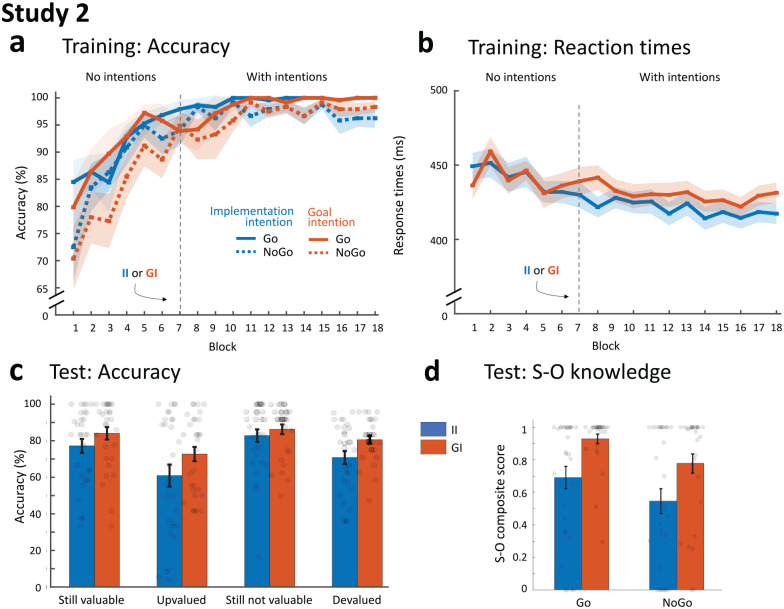
Main results of Study 2. (a) Mean accuracy across training. Similar to Study 1, participants learned to perform the task very well, both when using implementation and goal intentions, and significantly better for Go compared with NoGo stimuli. (b) Mean reaction times across training. Response times decreased over training and participants were faster when using implementation intentions. (c) Mean accuracy during test. In addition to the expected main effect of congruence, reflecting stimulus-driven habits, there was an even stronger (although unspecific) effect of implementation intentions compared with Study 1: performance was worse overall for stimuli trained with implementation compared with goal intentions. (d) Mean S-O knowledge. Consistent with Study 1, the use of implementation intentions (II) led to worse S-O knowledge then the use goal intentions, and non-valuable (NoGo) stimuli were also associated with lower SO-knowledge than valuable (Go) stimuli. Shaded regions (in A and B) and error bars (in C and D) represent standard error of the mean. II: implementation intention group; GI: goal intention group; RT: reaction time; S-O knowledge: Stimulus Outcome knowledge.

The training results are shown in Figure 3a and b. As expected, the repeated measures ANOVA with factors Block-set, Value and Intention showed that participants learned to make correct responses over the first part of training (main effect of block-set: *F*_3.6, 102.9_ = 30.35, *p* < .001 *η*_p_^2^ = .51). There was a significant main effect of value (*F*_1,29_ = 5.18, *p* = .03, *η*_p_^2^ = .15), driven by overall better performance on Go trials. As to be expected, there were no differences in accuracy for training with stimuli subsequently trained with implementation or goal intentions (all *p* > .23).

Response times for Go trials also decreased over training (*F*_1,29_ = 4.86, *p* < .001, *η*_p_^2^ = .14; Block-set 1: *M* = 443 ms, 95% CI = [432, 455]; Block-set 6: *M* = 434, 95% CI = [422, 446]).

#### Instrumental training with strategic planning

Another repeated measures ANOVA (Block-set by Value by Intention) revealed that participants continued to improve performance after intentions were introduced (main effect of block: *F*_1.9,53.7_ = 3.93, *p* = .03, *η*_p_^2^ = .12) and respond more accurately overall on Go compared with NoGo trials (main effect of value: *F*_1,29_ = 21.38, *p* < .001, *η*_p_^2^ = .42). Moreover, a borderline significant interaction between intention and block (*F*_2.2,61.7_ = 2.96, *p* = .056, *η*_p_^2^ = .09) was driven by significantly better performance with implementation intentions compared with goal intentions on the first block-set (*F*_1,29_ = 6.27, *p* = .02, *η*_p_^2^ = .18)—an effect that was absent in the later training block-sets (all *p* > .16, *F* < 2.0).

Furthermore, in addition to participants decreasing response times over the course of training on Go trials (*F*_3.4,98.7_ = 3.10, *p* = .03, *η*_p_^2^ = .10), using implementation intentions led to significantly faster responding across all training blocks than goal intentions (*F*_1,29_ = 13.82, *p* < .001, *η*_p_^2^ = .32).

Based on the results of the between-subjects study, we exploratively tested the direction of the effects of both intentions early in training (i.e., Block-set 6 vs Block-set 7). Although the pattern was similar as in the first study, that is, accuracy increased for implementation but decreased for goal intentions, these effects were not significant (*t* =–1.18, *p* = .25; and *t* = 1.11, *p* = .28, respectively).

#### Symmetrical outcome-revaluation test

The results of the test-phase are shown in Figure 3c. As expected, and in line with the results of Study 1, we observed a main effect of congruence (*F*_1,29_ = 24.95, *p* < .001, *η*_p_^2^ = .46) on the Test-value by Congruence by Intention repeated measures ANOVA. More importantly, there was a main effect of intention (*F*_1,29_ = 15.38, *p* < .001, *η*_p_^2^ = .35) reflecting significantly worse performance across congruent and incongruent trials for discriminative stimuli trained with implementation intentions compared with goal intentions—an effect that was strongly supported by Bayesian evidence (BF_incl_ = 12.1) and, contrary to Study 1, was seen across both Go and NoGo trials (no intention by value interaction: *F*_1,29_ = .43, *p* = .52, *η*_p_^2^ = .02, BF_excl_ = 2.59). Importantly, as in Study 1, the expected interaction between congruence and intention failed to reach significance (*F*_1,29_ = 2.26, *p* = .14, *η*_p_^2^ = .07), although the Bayesian evidence against an interaction was inconclusive (BF_excl_ = 1.04).

A similar analysis of response times did not yield any significant results (all *p* > .32), apart from a borderline significant effect of intention-type (*F*_1,19_ = 4.05, *p* = .059, *η*_p_^2^ = .18) due to faster responses for implementation intentions during test, in line with their effect during training.

#### S-O knowledge

S-O knowledge is shown in Figure 3d. The Value by Intention repeated measures ANOVA revealed that S-O knowledge was significantly lower for implementation (median = 56.5%, *SD* = 33.0) than for goal (median = 92.6%, *SD* = 18.7) intentions (*F*_1,29_ = 17.0, *p* < .001, *η*_p_^2^ = .37). Furthermore, explicit contingency knowledge was lower for stimuli associated during training with non-valuable than with valuable outcomes (*F*_1,29_ = 7.44, *p* = .01, *η*_p_^2^ = .20).

We again also explored the relationship between test-accuracy and S-O knowledge, which showed significant positive correlations for both conditions (implementation intentions: *r*_τ_ = .56, *p* < .001, 95% CI = [.39, .73]; goal intentions: *r*_τ_ = .35, *p* = .008, 95% CI = [.13, .57]).

### Interim conclusions Study 2

To summarise, in Study 2, we generally replicated the results of study 1. First, we found that strategic if-then planning gave a modest but significant boost to performance compared with goal intentions during instrumental learning. Importantly, by the end of instrumental training, discriminative performance was at an equal near-perfect level on goal and implementation intention trials. Conversely, implementation intentions *impaired* performance during the test phase when some of the discriminative stimuli signalled revalued outcomes, requiring flexible adjustment of the learnt response. While we had predicted that implementation intentions would increase the magnitude of the congruence effect (i.e., lead to worse performance on incongruent [devalued and upvalued] relative to congruent [still not valuable and still valuable] trials), we observed an impairment across all trial types. Although not in line with our preregistered hypothesis, these results were less surprising when considering the results of Study 1 where we observed a similar effect of implementation intentions across congruent and incongruent trials, albeit restricted to Go trials (still valuable and upvalued trials).

## General discussion

To determine whether if-then plans, known as implementation intentions, create “instant habits” by strengthening stimulus-response (S-R) associations, we conducted two experiments with an outcome-revaluation paradigm. We found that implementation (compared with goal) intentions initially led to better performance during instrumental training, in terms of accuracy (in both studies) and speed (in Study 2). This beneficial effect of strategic if-then planning on instrumental learning is in line with previous evidence for increased efficiency or automaticity (Bieleke et al., 2021; Brandstatter et al., 2001; Parks-Stamm et al., 2007). Crucially, to test whether this increased efficiency comes at the cost of reduced flexibility—a hallmark of habitual behaviour—we tested whether participants could overcome the learnt stimulus-response mapping when some of the signalled outcome values changed (i.e., were revalued). In Experiment 1, we found that participants in the implementation intention condition performed worse than the goal intention group, but only when a motor (Go) response was required (i.e., the outcome was valuable during test). In Experiment 2, we conducted a within-subject manipulation of strategic planning, and this time the detrimental effect of if-then planning was even more robust: across all trial types, participants performed worse when they had used implementation intentions to stamp in the required S-R mapping during training relative to goal intentions. Importantly, the detrimental effect of implementation intentions in both experiments was not dependent on whether the required S-R mapping changed during test due to revaluation of the outcome (i.e., it was observed across congruent and incongruent trials), suggesting that this planning strategy failed to instal “instant habits” by strengthening S-R associations. Rather, it appears that implementation intentions impaired the ability to perform in a goal-directed manner when goals changed by (initially) diminishing the focus on outcome learning.

These results extend the findings by Legrand et al. (2017), who previously investigated if implementation intentions lead to perseverance in the face of adverse consequences (by providing punishments). A critical difference in our study design is that we conducted an outcome-revaluation test in the absence of feedback (extinction) to prevent new instrumental learning to occur. This allowed us to test for “action slips”: failures to immediately and flexibly adjust behaviour when outcome values change. We expected the use of implementation intentions to result specifically in more action slips, but instead we found a more generally impairing effect on test performance. Thus, our findings extend (Legrand et al., 2017) by showing that, in the absence of new learning, if-then planning leads to inflexibility when outcome values change. But how can we explain this general detrimental impact of implementation intentions on test performance (instead of on incongruent trials specifically)? In our view, the most plausible explanation is that using this S-R planning strategy during instrumental learning may have focused attention and reliance on S-R mappings, thereby shifting attention and learning away from the outcomes compared with goal intentions. In turn, this may have impaired the ability to accurately predict the available outcome, which is vital for goal-directed action control. Although S-O knowledge is arguably more crucial for incongruent than for congruent trials, our test phase may have encouraged a goal-directed strategy towards all trial types, regardless of whether the signalled outcome value had changed. As a result, performance on congruent trials could also have suffered from impaired S-O knowledge. In line with this possibility, implementation intentions led to reduced knowledge of the S-O contingencies in both experiments, as measured by questionnaires at the end of training. Furthermore, individual differences in S-O knowledge were positively associated with overall accuracy in the test phase.

Our findings are also relevant in the context of a broader discussion in the habit literature regarding the role of dual processes (Watson & de Wit, 2018)—specifically, the question whether variations in habit strength independently contribute to behavioural flexibility, or whether this is predominantly determined by variations in goal-directed control. The current findings suggest that the detrimental impact of S-R planning is mediated by reduced S-O learning, thus impairing the ability to act in a goal-directed manner under unstable conditions when some outcomes change in value, rather than by impacting an independent habit process. It follows that when S-O knowledge is already perfect (due to very simple contingencies, salient outcomes, or preexisting S-O knowledge prior to action planning) implementation intentions have the benefit of efficiency while still allowing for flexible action control, although this remains to be determined by future research.

Based on the current findings, it is difficult to determine whether implementation intentions had an adverse effect on test performance or goal intentions had a beneficial effect, or indeed both. Specifically, it is possible that (some) participants use implementation-intention-like strategies spontaneously when performing this task (Verhoeven et al., 2018), by focusing on the required S-R mappings. If this is the case, then goal intentions may have hindered this spontaneous strategy (as opposed to implementation intentions encouraging S-R learning). While this may have led to an initial reduction in accuracy and reaction time during the learning phase, where the S-R mappings were most crucial, this would improve performance during the test phase where knowledge of the S: R-O relationships is vital. Our results seem to suggest a combination of both, as performance improved after the introduction of implementation but declined after goal intentions compared with the last block before the introduction of intentions. Taking this argument even further, the spontaneous application of strategic S-R planning on experimental tasks like the one we have employed here may actually lead to people to rely on S-R strategies much more quickly than previously assumed, leading to a rapid instead of gradual transition from goal-directed to habitual behaviour. This may hamper the investigation of habit formation as a function of extensive training: if participants switch to a S-R strategies early on, this will limit the additional effect of longer training—which might even offer an explanation for reported difficulties of recent studies in showing effects of behavioural repetition on performance (de Wit et al., 2018; Watson et al., 2022). In future research, the impact of spontaneous action planning strategies on outcome revaluation performance could be determined by adding a control condition without instructed action planning, and comparing that to planning with goal and implementation intentions. Relatedly, the fact that we found a more pronounced effect of implementation intentions in Study 2 than in Study 1 may have to do with the fact that, *per intention type*, participants received only half the amount of training with intentions. On one hand, an accelerated transition from goal-directed performance based on anticipated outcomes (via S-O associations) towards reliance on S-R habits may be achieved through the use implementation intentions. However, this transition may also take place with repeated instrumental performance. More extensive training could therefore mask any additional effect of strategic planning. Following this logic, the biggest effect of if-then planning may be observed early on and shortening the amount of instrumental training with strategic planning may enhance the effect of implementation intentions on test-performance. It would therefore be interesting for future research to test the effect of implementation intentions with shorter instrumental training. Another way to obtain a better understanding of the role of (potentially) dual processes underlying if-then planning and its effects on instrumental action control is by examining the underlying neural dynamics. Findings from previous EEG studies are in line with the notion that implementation intentions—like S-R habits—exert their effect faster than the indirect goal-directed pathway (for a review, see Wieber et al., 2015). One previous fMRI study investigated whether the enactment of an implementation (versus goal) intention during a prospective memory task action had differential effects on action control and associated brain activity (Gilbert et al., 2009), but neuroimaging research directly investigating the effects of implementation intentions on flexibility is lacking. An open question therefore remains whether using implementation intentions activates similar corticostriatal regions as implicated in goal-directed and habitual actions (Balleine & O’Doherty, 2010). Future research should use an outcome-revaluation task with implementation intentions during neuroimaging to examine the underlying neural mechanisms. Using a well-controlled outcome-revaluation paradigm, we provided evidence for the beneficial effects of implementation intentions on accuracy and speed during instrumental learning, in line with previous evidence for their beneficial effects (Bieleke et al., 2021), while also providing insight into the processes underlying implementation intentions and behavioural flexibility. However, the question remains whether our findings with this artificial computerised task can be generalised to real-life action planning. Does the use of implementation intentions in everyday situations also lead to deficient learning of complex contingencies, and consequently less flexible behaviour when a strategy needs to be adjusted? As it stands, our findings warrant caution for applying if-then planning to situations where the agent does not possess perfect knowledge of complex, behavioural (motor-)contingencies with high time pressure that sometimes require flexibility, such as sports, aviation, or surgery. For example, in soccer, coaches may apply if-then planning to improve players’ recognition and application of complex tactical situations through S-R learning. However, if players consequently do not learn the consequences of these automatically triggered actions, this may lead to problems in flexibly adjusting when faced with an opponent that uses counter-tactics that interfere with these predefined action-plans. This potential detrimental effect of implementation intentions should be investigated in future research in real-life settings.

In conclusion, this study was inspired by the original suggestion that if-then plans create a strong associative link between the specified opportunity to act (cue) and the response, which leads to the response being elicited automatically (Gollwitzer, 1999). In turn, the effectiveness of if-then planning is mediated via the same S-R mechanism that underlies habit formation as a consequence of extensive behavioural repetition. Our study is the first to formally test the “instant habit” account of implementation intentions using an experimental paradigm designed to test the contribution of dual (habitual and goal-directed) processes to action control. Our results suggest that implementation intentions stimulate the efficient initiation of a planned response during stable conditions (i.e., when simply executing the planned response during the learning phase), but under unstable conditions have a negative impact on behavioural control. This detrimental effect on behavioural flexibility is not a result of stronger S-R associations or “instant habits,” but rather of reduced learning about behavioural outcomes and consequently a lack of goal-directed control.
